# The relationship between the network of non-coding RNAs-molecular targets and N6-methyladenosine modification in tumors of urinary system

**DOI:** 10.1038/s41419-024-06664-z

**Published:** 2024-04-17

**Authors:** Ruiming Li, Chunming Zhu, Yuan Wang, Xia Wang, Yibing Wang, Jiahe Wang, Kefeng Wang

**Affiliations:** 1 Department of Urology, Shengjing Hospital of China Medical University, Shenyang, 110004 China; 2 Department of Family Medicine, Shengjing Hospital of China Medical University, Shenyang, 110004 China; 3 Department of General Surgery, Shengjing Hospital of China Medical University, Shenyang, 110004 China

**Keywords:** Urological cancer, Non-coding RNAs

## Abstract

N6-methyladenosine (m6A) methylation, a prevalent eukaryotic post-transcriptional modification, is involved in multiple biological functions, including mediating variable splicing, RNA maturation, transcription, and nuclear export, and also is vital for regulating RNA translation, stability, and cytoplasmic degradation. For example, m6A methylation can regulate pre-miRNA expression by affecting both splicing and maturation. Non-coding RNA (ncRNA), which includes microRNAs (miRNAs), long non-coding RNAs (lncRNAs), and circular RNAs (circRNAs), does not encode proteins but has powerful impacts on transcription and translation. Conversely, ncRNAs may impact m6A methylation by affecting the expression of m6A regulators, including miRNAs targeting mRNA of m6A regulators, or lncRNAs, and circRNAs, acting as scaffolds to regulate transcription of m6A regulatory factors. Dysregulation of m6A methylation is common in urinary tumors, and the regulatory role of ncRNAs is also important for these malignancies. This article provides a systematic review of the role and mechanisms of action of m6A methylation and ncRNAs in urinary tumors.

## Facts

- M6A regulators play important roles in urinary system tumors.
- There are significant interactions between m6A modification and ncRNAs in urinary system tumors.
- The research of pathogenesis between m6A modification and ncRNAs is of great significance for the development of specific targeted drugs.

## Open questions

- What is the impact of m6A modification and ncRNAs in the development of urinary system tumors?
- What is the best approach to further investigate the interaction between m6A modification and ncRNAs to promote anti-tumor immunity in urinary system tumors?
- Can the interaction between m6A modification and ncRNAs be used as biomarkers to predict chemotherapy resistance in urinary tumors therapies?

## Introduction

Malignant tumors of the urinary system include prostate cancer (PCa), bladder cancer (BCa), and renal cell carcinoma (RCC). In the United States, PCa had the highest incidence among all male cancers in 2023, with 29% of 1,010,310 total cancers in men. BCa accounted for 62,420 cases (6%), while RCC accounted for 52,360 cases (5%) [1]. Conventional urinary tumor treatment typically begins with surgical resection, which often provides a good prognosis. However, surgical treatment is insufficient for patients with advanced disease. Therefore, a detailed examination of the mechanisms affecting tumor progression is of great significance for developing novel therapies and improving patient prognosis.

N6-methyladenosine (m6A) was first described in 1974 and is typically regulated by m6A writers, readers, and erasers [2]. Most m6A modifications occur in the 3′ untranslated region (3′UTR), the region of the stop codon, and internal long exon regions. M6A methylation impacts tumor proliferation, invasion, and metastasis by regulating the cleavage, transport, stability, and degradation of RNAs [3]. For example, METTL14 can induce sunitinib resistance in ccRCC by enhancing the stability of tumor necrosis factor receptor-associated factor 1 mRNA and promoting its expression [4]. Liu et al. [5] showed that METTL14-mediated m6A modification promoted tumor progression in BCa cells by promoting lncDBET expression, which then directly interacted with FABP5 to activate the PPAR pathway to promote lipid metabolism.

Non-coding RNAs (ncRNAs), including microRNAs (miRNAs), long non-coding RNAs (lncRNAs), and circular RNAs (circRNAs), account for more than 90% of the total transcriptional output of human cells [6, 7]. With the development of advanced sequencing technology, increasing numbers of ncRNAs have been found to play important roles in tumor progression, regulating mRNA transcription and protein translation. For example, miR-1 can affect tumor progression by targeting CXCR4/FOXM1/RRM2 in small-cell lung cancer patients [8]. LncRNA AY can bind to the ITGAV promoter region to remodel its chromatin structure and recruit RNA polymerase II to promote mRNA transcription, ultimately promoting hepatocellular carcinoma migration [9]. Hsa_circ_0076611 promotes triple-negative breast cancer cell proliferation by interacting with the mRNA of multiple proliferation-related genes, binding to the translation initiation mechanism and promoting protein expression [10]. Most cancer are induced by gene mutations in non-coding regions, but gene therapy targeting ncRNAs is rarely used clinically, most probably because of its recent discovery and uncertain mechanism of action [11]. Therefore, studying the mechanisms by which ncRNAs influence cancer progression, including their interaction with m6A modifications, is of great significance for the development of new gene therapy drugs.

To better study the mechanism by which urinary tumors occur and develop, the role of m6A and ncRNAs in urinary tumors is systematically reviewed in this paper, suggesting approaches for the development of new targeted therapies.

## Overview of m6A regulators

**Fig. 1:**
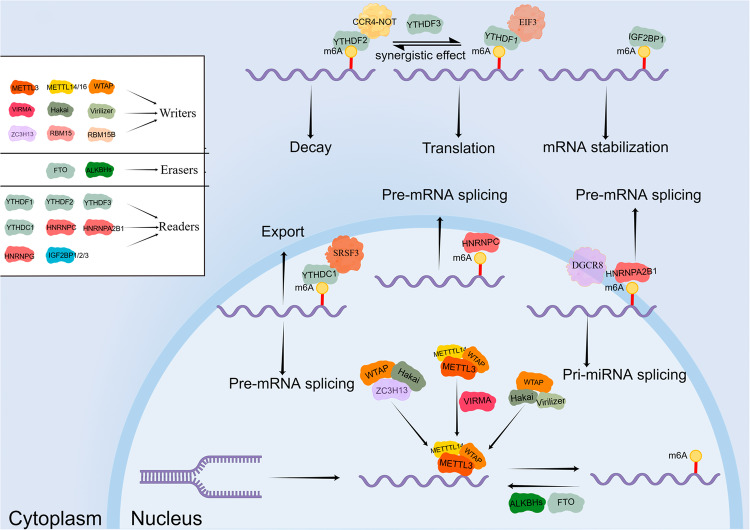
Functions of m6A regulators. M6A “writers” include METTL3, METTL14/16, WTAP, VIRMA, Hakai, Virilizer, ZC3H13, RBM15, and RBM15B; M6A “erasers” include FTO and ALKBHs; M6A “readers” include YTHDF1, YTHDF2, YTHDF3, YTHDC1, HNRNPC, HNRNPA2B1, HNRNPG, and IGF2BP1/2/3. Their functions are involved in RNA degradation, stabilization, and translation, pre-mRNA splicing, and transport, etc.

The m6A regulators include “writers”, “erasers”, and “readers” [12]. M6A modification plays a role in mRNA splicing, processing, translation, stability, nuclear export, decay, and other processes (Fig. 1) [13], and also regulates the stability and degradation of miRNAs, lncRNAs, and circRNAs, modulating biological processes, including tumor progression, migration, immunity, and drug resistance [14, 15].

### The m6A writers

M6A methyltransferases include METTL3, METTL14, METTL16, Wilms Tumor 1 associated protein (WTAP), Virilizer, Hakai, ZC3H13, Vir-like m6A methyltransferase associated (VIRMA), RNA-binding motif protein 15 (RBM15), and RBM15B.

#### The METTL family

The METTL family is the most common group of methyltransferases. METTL3, the core enzyme of m6A methyltransferase, is an S-adenosine methionine binding protein and is the only catalytic subunit of m6A MTC, which can bind about 22% of m6A sites [16]. METTL14, the second supporting enzyme, co-localizes with METTL3 in the nuclear speckle, forming a stable heterodimer in a ratio of 1:1. METTL16 can catalyze m6A generation on U6 small nuclear RNA to target pri-mRNAs and ncRNAs [17]. METTL16 can also catalyze the formation of m6A in the conserved hairpin sequence of MAT2A mRNA, thereby regulating S-adenosine methionine enzyme synthesis homeostasis [18]. The METTL family plays an important role in cancers, for example, METTL3 induces small-cell lung cancer chemoresistance through causing destabilization of Decapping Protein 2 RNA by m6A methylation modification [19]. METTL14 inhibits the metastasis of colorectal cancer through m6A modification of SRY-related high-mobility-group box 4 [20]. Dai et al. [21] reported that METTL16 could regulate the stability of lncRNA RAB11B-AS1 through m6A modification to affect the proliferation, migration, and invasion of hepatocellular carcinoma.

#### WTAP and other coenzyme factors

WTAP, as a shear factor, guides the location of the METTL14-METTL3 complex in the nuclear speckle of pre-mRNA and affects m6A methylation [22–24]. The Virilizer and Hakai complex binds WTAP to promote m6A methylation in mammalian cells [25]. In addition, Wen et al. [26] demonstrated that ZC3H13 promotes nuclear m6A methylation generation by binding Virilizer, Hakai, and WTAP. Yue et al. [27] showed that VIRMA can recruit the catalytic core factor METTL3/METTL14/WTAP to promote region-selective methylation, and some 60% of VIRMA’s immunoprecipitation targets demonstrated m6A enrichment in 3′UTR and near the stop codon. RBM15 and its paralogue RBM15B recruit the m6A complex to specific RNA sites, mediating methylation [28]. WTAP-mediated m6A modification significantly increased the stability of lncRNA DIAPH1-AS1 to regulate tumor growth and metastasis in nasopharyngeal carcinoma.

These results suggest that m6A methyltransferases are vital for RNA m6A methylation, with METTL3 exhibiting the highest level of m6A methylase activity. Given their interactions, these enzymes often form complexes that promote modulation to m6A on their targets.

### The m6A erasers

M6A demethylases include fat mass and obesity-associated protein (FTO) and ALKB homologous (ALKBHs), which belong to a class of dioxygenases that rely on Fe2+ and α-ketoglutaric acid as cofactors, causing m6A demethylation through oxidative dealkylation [29].

#### FTO

The identification of FTO, the earliest m6A demethylation transferase discovered, suggested that m6A methylation is a dynamic and reversible regulatory process [30]. FTO affects adipocyte differentiation by regulating the level of m6A, impacting RUNX1T1 exon splicing [31]. FTO can also reduce the stability of APOE mRNA to inhibit glycolytic metabolism in papillary thyroid carcinoma [32]. Moreover, FTO functions as an oncogene in acute myeloid leukemia by regulating m6A methylation levels, and regulating the expression of ASB2 and RARA [33].

#### The ALKBH family

ALKBH5, as the second demethylated transferase identified, significantly impacts mRNA output and mRNA metabolism in nuclear spots [34]. ALKBH5 can inhibit distant metastasis and lymph node metastasis in gastric cancer by attenuating m6A modification of PKMYT1, reducing its stability [35]. Moreover, ALKBH6 and ALKBH9C function as m6A demethylases in Arabidopsis [36, 37].

In conclusion, the discovery of m6A demethylation deepens the understanding of m6A methylation and confirms that demethylases and methyltransferases play complementary roles in the dynamic, reversible processes of m6A modification.

### The m6A readers

The major m6A readers are the evolutionarily conserved YT521-B homology (YTH) family, the heterogeneous nuclear ribonucleoprotein (HNRNP) family, and the IGF2BP family. These include YTHDF1, YTHDF2, YTHDF3, YTHDC1, HNRNPC, HNRNPA2B1, HNRNPG, and IFG2BP(1/2/3), with high affinity for domains containing m6A [38].

#### The YTH family

M6A readers specifically identify the binding sites of m6A, determine the m6A modification, and then regulate the downstream gene expression and biological function. YTHDF2, the first m6A reader identified, participates in the dynamic regulation of mRNA 5′UTR methylation by restricting FTO [39]. Du et al. [40] showed that YTHDF2 degraded m6A-modified RNA by recruiting the deadenylase complex CCR4-NOT. YTHDF2 can destabilize the lncRNA XIST by recognizing m6A methylation sites [41]. In contrast, in the loop structure of EIF4G, YTHDF1 promotes translation initiation and protein synthesis by recruiting m6A-modified transcripts through its interaction with eIF3 [42]. Shi et al. [43] reported that YTHDF3 and YTHDF1 function together to promote protein synthesis and methylated mRNA decay through YTHDF2. Xu et al. [44] identified the crystal structures of the YTH domain of YTHDC1, a member of the YTH domain family, complexed with an m6A-containing RNA. YTHDC1 promotes mRNA splicing, nuclear specular localization, and RNA binding by specifically binding with mRNA clipping factor SRSF3. However, YTHDC1 loses these functions in the absence of m6A, suggesting that YTHDC1 regulates mRNA splicing by acting as an m6A reader [45].

#### The HNRNP family

Members of the HNRNP family also function as m6A readers. HNRNPC, an RNA binding protein, processes pre-mRNA through binding RNA binding motifs impacted by RNA structural changes induced by m6A [46]. HNRNPA2B1 binds to m6A marks in nuclear transcripts, promoting primary miRNA (pri-miRNA) splicing through its interaction with DGCR8, a microRNA microprocessor protein [47]. Liu et al. [48] demonstrated that HNRNPG binds to m6A-modified RNA through its C-terminal low-complex region, thereby affecting downstream biological function changes.

#### The IGF2BP family

Some studies have revealed that insulin-like growth factor 2 mRNA-binding proteins (IGF2BPs; including IGF2BP1/2/3), as a unique family of m6A readers, can target numerous diverse mRNA transcripts through recognition of consensus GG(m6A)C sequences [49]. For example, IGF2BP1 recognizes METTL3-modified JAK2 mRNA methylation sites, improving mRNA stability, thereby increasing expression [50]. IGF2BP1 affects transcriptional regulation by altering the expression of SRF in several cancers, causing a worse prognosis [51]. In a similar fashion, IFGBP2 promotes metastasis and angiogenesis in lung adenocarcinoma by recognizing FLT4 m6A modification, increasing its mRNA stability [52]. IGF2BP3 inhibits immune surveillance of breast cancer by recognizing m6A modification on PDL-1 and enhancing its mRNA stability.

In summary, m6A readers bind to specific m6A sites, thereby influencing RNA metabolism and determining downstream biological effects.

## The interaction between m6A modification and ncRNAs in urinary tumors

### The interaction between m6A modification and ncRNAs in PCa

PCa is the most common cancer in men, with bone metastases occurring, predominantly in the hip, pelvis, and spine, in about 80% of patients with advanced PCa. The 5-year survival for metastatic PCa is only 3% [53, 54], and androgen deprivation therapy is the most common therapy for PCa. However, after a period of treatment, some patients develop more aggressive CRPC, which has an average survival of only 1.5 years [55, 56]. M6A modification of ncRNAs regulates multiple biological functions by altering gene expression [15], which can modulate PCa cell proliferation and survival and play a crucial role in PCa [57]. Therefore, further evaluation of the interaction between m6A modification and ncRNAs in PCa is needed.

#### Promoting the cleavage and maturation of pri-miRNA in PCa

**Fig. 2:**
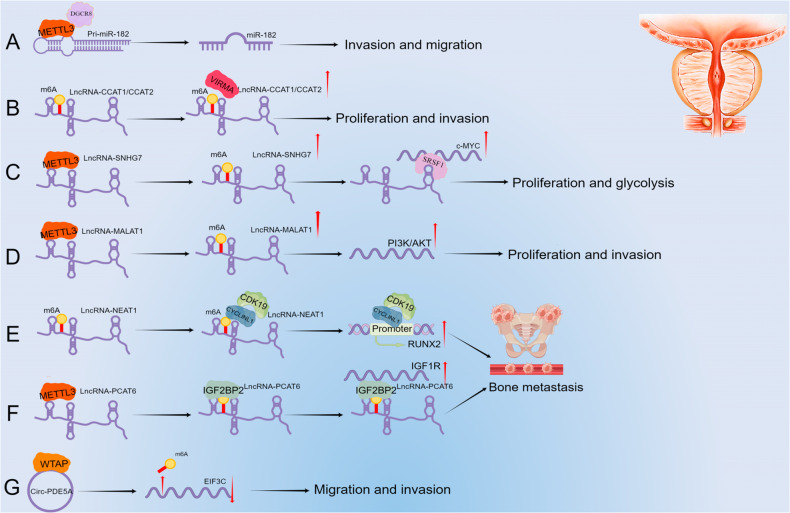
The interactions between m6A methylation and ncRNAs in PCa. **A** METTL3 bound to DGCR8, identified the m6A site on pri-miR-182, promoted its maturation, and ultimately accelerated PCa invasion and migration. **B** VIRMA stabilized the expression of lncRNA CCAT1/CCAT2 by targeting its m6A site and promoted the proliferation and invasion of PCa. **C** METTL3 stabilized the expression of lncRNA SNHG7 through m6A modification, facilitated its binding with SRSF1, and ultimately increased the expression of c-MYC, thus promoting the proliferation and glycolysis of PCa. **D** METTL3 promoted the expression of lncRNA MALAT1 through m6A modification, thereby affecting the PI3K/AKT pathway and promoting the proliferation and invasion of PCa. **E** LncRNA NEAT1 can recruit CYCLINL1 and CDK19 to the promoter region of RUNX2 through m6A modification sites and promote PCa bone metastasis. **F** METTL3 can mediate lncRNA PCAT6 m6A modification, and then bind IGF2BP2 and IGF1R mRNA to form a complex to stabilize its expression, and ultimately promote PCa bone metastasis. **G** WTAP formed a complex with circPDE5A, bound to EIF3C, and ultimately down-regulated its expression, promoting PCa migration and invasion.

M6A modification can promote pri-miRNA cleavage and maturation. METTL3 is highly expressed in PCa, resulting in high m6A methylation levels [58]. Multiple studies have confirmed that miR-182 is involved in the progression of PCa [59, 60]. Wang et al. [61] found that METTL3 promotes m6A methylation on pri-miR-182, the precursor of miR-182, and subsequently interacts with DGCR8 to promote its cleavage and maturation, leading to increased miR-182 expression, promoting PCa invasion and migration (Fig. 2A).

#### Enhancing the stability of ncRNAs in PCa

M6A modification increases ncRNA expression by enhancing its stability. VIRMA promotes the expression of lncRNA CCAT1 /CCAT2 in an m6A-dependent manner, thereby accelerating PCa cell proliferation and invasion (Fig. 2B) [62]. Liu et al. [63] reported that METTL3 stabilizes lncRNA SNHG7, which interacts with SRSF1 to influence the expression of c-MYC and promote the proliferation and glycolysis of PCa cells (Fig. 2C). Mao et al. [64] showed that METTL3 promotes the expression of lncRNA MALAT1 by increasing its m6A modification, facilitating PCa proliferation and invasion by regulating the PI3K/AKT pathway (Fig. 2D).

#### Promoting the transcription of ncRNAs in PCa

M6A modification can increase ncRNA levels by promoting their transcription. For example, Wen et al. [65] found that the lncRNA NEAT1 can bind to CYCLINL1 through the m6A site, increasing the activity of the CYCLINL1/CDK19 complex, then binding to the RUNX2 promoter to promote the transcription of RUNX2, eventually leading to PCa malignant progression and bone metastasis (Fig. 2E). Similarly, Lang et al. [66] reported that the lncRNA PCAT6 has multiple m6A sites, and showed that METTL3 regulates RNA stability by affecting IGF2BP2 binding and recognition of methylated PCAT6. The downstream impact of this methylation was to enhance the stability of IGF1R mRNA by directly binding IGF1R mRNA, and ultimately regulate PCa malignant progression and bone metastasis (Fig. 2F). Another research group showed that circPDE5A inhibited the methylation of EIF3C mRNA by forming a complex with WTAP, resulting in decreased expression of EIF3C, inactivation of MAPK pathway, and inhibition of migration and invasion of PCa cells (Fig. 2G) [67].

Taken together, these studies suggest that METTL3 can promote the cleavage and maturation of pri-miR-182 through m6A modification in PCa. Secondly, VIRMA and METTL3 can enhance the stability of lncRNA CCAT1/CCAT2, lncRNA SNHG7, and lncRNA MALAT1 through m6A modification, and thus increasing their expression in PCa. Finally, METTL3, IGF2BP2, and WTAP can promote the transcription of lncRNA NEAT1, lncRNA PCAT6, and circPDE5A, respectively, through m6A modification, and improve their expression in PCa.

### The interaction between m6A modification and ncRNAs in BCa

BCa is the fifth-most common malignancy in the world and the second-most common cancer of the urinary system. Approximately 70% of patients with BCa have non-muscle-invasive BCa, and the 5-year survival rate for them can be as high as 85% after transurethral resection of the bladder tumor. However, with muscle-invasive BCa, the 5-year survival rate is low [68]. Further study of the pathogenesis of BCa is crucial to improve the survival rate of patients with BCa.

#### Promoting the cleavage and maturation of pri-miRNAs in BCa

**Fig. 3:**
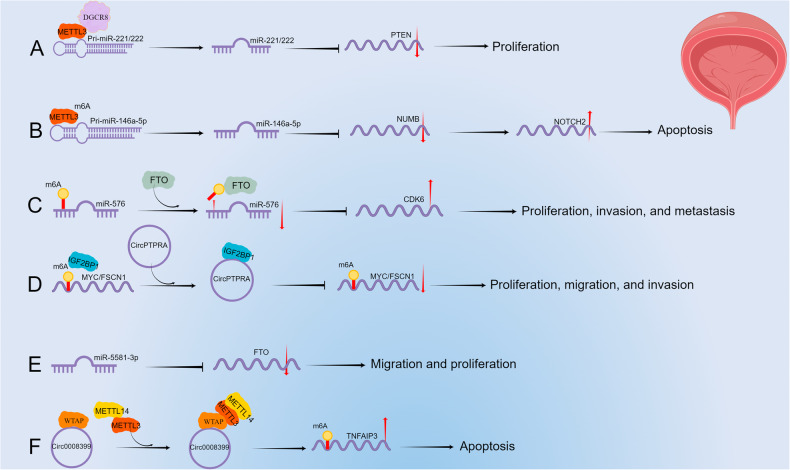
The interactions between m6A methylation and ncRNAs in BCa. **A** METTL3 bound to DGCR8, identified the m6A site on pri-miR-221/222, promoted its maturation, and ultimately inhibited the expression of PTEN to stimulate the proliferation of BCa. **B** METTL3 promoted the maturation of pri-miR-146a-5p, and then affected the NUMB/NOTCH2 pathway to inhibit BCa apoptosis. **C** FTO decreased the expression of miR-576 by removing m6A methylation on miR-576, and finally promoted the expression of CDK6, and stimulated the proliferation, invasion, and metastasis of BCa. **D** CircPTPRA bound to IGF2BP1 and blocked its binding to MYC/FSCN1, leading to a decrease in its expression level and promoting BCa apoptosis. **E** MiR-5581-3p attenuated BCa migration and proliferation by acting as a sponge to inhibit FTO expression. **F** Circ0008399 promoted the methylation of m6A on TNFAIP3 mRNA by binding to WTAP/METTL3/METTL14 complex, and eventually promoted its expression, leading to BCa apoptosis.

M6A modification can promote pri-miRNA cleavage and maturation. Han et al. [69] showed that METTL3 can promote the binding of miR-221/222 to DGCR8 by affecting the methylation level of m6A in pri-miR-221/222, ultimately leading to its maturation, inhibiting the expression of PTEN, and leading to BCa proliferation (Fig. 3A). In addition, Yan et al. [70] demonstrated that the honey bee venom component melittin inhibits the maturation of pri-miR-146a-5p, thereby promoting downstream NUMB expression, leading to the inactivation of the NOTCH2 pathway, inducing apoptosis. However, this effect can be blocked by overexpression of METTL3, impacting m6A modification to promote the maturation of pri-miR-146a-5p (Fig. 3B). Zhou et al. [71] reported that FTO regulates pri-miR-576 through m6A methylation and affects its downstream target gene CDK6 to promote BCa proliferation, invasion, and metastasis (Fig. 3C).

#### Influencing the expression and function of m6A regulators in BCa

As an example of counter-regulation, ncRNAs can affect the expression and function of m6A regulators. For example, circPTPRA, by binding to the KH domain of IGF2BP1 in the cytoplasm, blocks IGF2BP1 from recognizing the m6A modification sites of the downstream genes MYC and FSCN1, inhibiting their expression, and ultimately suppressing BCa cell proliferation, migration, and invasion (Fig. 3D) [72]. Sun et al. [73] reported that miR-5581-3p can inhibit the migration of BCa cells by targeting FTO (Fig. 3E). Wei et al. [74] showed that circ0008399 promotes the formation of the WTAP/METTL3/METTL14 complex, increasing TNFAIP3 mRNA m6A methylation to enhance its mRNA stability, inhibiting BCa cell apoptosis, thus promoting the resistance of BCa cells to carboplatin-dependent chemotherapy (Fig. 3F).

Overall, these data demonstrate that METTL3 promotes the cleavage and maturation of pri-miR-221/222, pri-miR-146a-5p, and pri-miR-576 through m6A modification in BCa. In addition, circPTPRA, miR-5581-3p, and circ0008399 influenced the expression and function of IGF2BP1, FTO, and the WTAP/METTL3/METL14 complex, respectively, in BCa.

### The interaction between m6A modification and ncRNAs in RCC

According to recent cancer statistics, RCC ranked sixth among men and ninth among women in 2023 [1]. With the increased availability of abdominal imaging, the incidence of RCC is also increasing; however, distant metastases still occur in 17% of patients [75]. Although the prognosis of RCC is good after radical surgical resection, the 5-year survival rate for metastatic RCC is only 11% [76, 77]. To prolong survival in advanced RCC, new therapeutic targets must be identified.

#### Enhancing the stability of ncRNAs in RCC

**Fig. 4:**
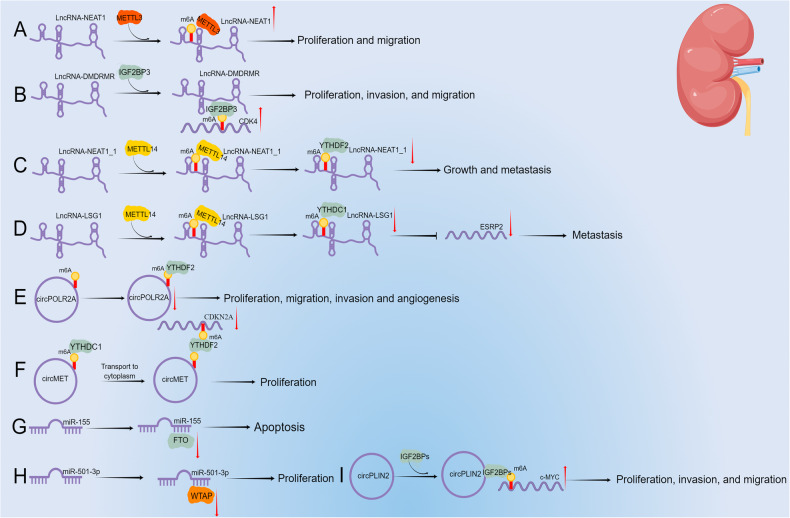
The interactions between m6A methylation and ncRNAs in RCC. **A** METTL3 bound to lncRNA NEAT1 to induce its m6A methylation and promote its expression, ultimately boosting the proliferation and migration of RCC. **B** LncRNA DMDRMR promoted the proliferation and migration of RCC by binding to IGF2BP3 and CDK4. **C** METTL14 can bind to lncRNA NEAT1_1 to induce m6A methylation, and then combine with YTHDF2 to inhibit its expression, thereby restraining the growth and metastasis of RCC. **D** METTL14 can bind to lncRNA LSG1 to induce m6A methylation, and then combine with YTHDC1 to inhibit its expression, thereby retarding the metastasis of RCC. **E** YTHDF2 can inhibit circPOLR2A expression by binding to its m6A modification sites. **F** YTHDC1 bound to the m6A site on circMET, promoted its transport to the cytoplasm, and then bound to YTHDF2, inhibited the expression of CDKN2A, and finally promoted the proliferation of RCC. **G** MiR-155 inhibited RCC apoptosis by targeting FTO expression. **H** MiR-501-3p inhibited RCC proliferation by targeting WTAP and restraining its expression. (**I**) CircPLIN2 can promote the expression of c-MYC by binding to IGF2BPs, and finally promote the proliferation, invasion, and migration of RCC.

M6A modification can enhance ncRNA expression by enhancing its stability. Chen et al. [78] found that the expression of the lncRNA NEAT1 was decreased in RCC, and m6A methylation level was low. Transfection of CRIPSR/dCas13b-METTL3 increased m6A methylation, decreased RCC cell proliferation and migration, and increased NEAT1 expression (Fig. 4A). Gu et al. [79] showed that the lncRNA DMDRMR can identify m6A modification sites on the target genes by binding to IGF2BP3, enhancing the stability of CDK4 mRNA, and promoting RCC cell proliferation, invasion, and migration (Fig. 4B). In addition, Liu et al. [80] reported that METTL14 regulates lncRNA NEAT1_1 methylation levels, leading to recognition and degradation by YTHDF2, ultimately inhibiting RCC cell growth and metastasis (Fig. 4C). Similarly, Shen et al. [81] found that METTL14 regulates lncRNA LSG1 m6A methylation levels, leading to recognition and degradation by YTHDC1. METTL14 inhibited ccRCC metastasis through the METTL14/lncRNA LSG1/YTHDC1/ESRP2 axis (Fig. 4D). Xu et al. [82] reported that circPOLR2A was highly expressed in ccRCC and correlated with tumor size and TNM stage. In the cytoplasm, YTHDF2 inhibits ccRCC proliferation, migration, invasion, and angiogenesis by recognizing the m6A site on circPOLR2A and causing its degradation (Fig. 4E).

#### Influencing the nuclear translocation of ncRNA in RCC

M6A methylation can affect the function of ncRNA by affecting its nuclear translocation. Transfection of NONO-TFE3 in NONO-TFE3 RCC cells can promote circMET expression by binding to circMET promoter sites. Mechanistically, YTHDC1 recognized the m6A site on circMET and promoted its transport into the cytoplasm. Subsequently, its expression was inhibited and cell proliferation was affected by binding of CDKN2A mRNA and recruitment of YTHDF2 to CDKN2A transcripts (Fig. 4F) [83].

#### Influencing the expression and function of m6A regulators in RCC

NcRNAs can affect the expression and function of m6A regulators in RCC. Yang et al. [84] reported that miR-155 inhibition increases FTO expression by decreasing its targeted inhibition of FTO, thus reducing m6A levels, and ultimately inducing cell apoptosis (Fig. 4G). MiR-501-3p blocks RCC proliferation by targeting the expression of WTAP, leading to G1 cell cycle arrest (Fig. 4H) [85]. Zhao et al. [86] found that circPLIN2 binds to the KH domain of IGF2BPs to enhance the stability of c-MYC mRNA by means of m6A modification. CircPLIN2 binds competitively to miR-199a-3p, weakening the inhibitory effect of ZEB1, and promoting ccRCC cell proliferation, invasion, and migration (Fig. 4I).

In summary, METTL3, IGF2BP3, METTL14, and YTHDF2 enhance the expression in RCC of lncRNA NEAT1, lncRNA DMDRMR, lncRNA NEAT1_1, lncRNA LSG1, and circPOLR2A, respectively, by enhancing their stability through m6A modifications. Secondly, YTHDC1 and YTHDF2 influence the nuclear translocation of circMET in RCC. Finally, miR-155, miR-501-3p, and circPLIN2 influence the expression and function of FTO, WTAP, and IGF2BPs by means of m6A modifications in RCC.

The interactions between m6A regulators and ncRNAs are listed in Tables 1–2.

**Table 1:** Molecular mechanism and biological functions of m6A in ncRNAs.

Molecule	Mechanism	NcRNA	Biological function	Tumor	References
METTL3	Promote the maturation of pri-miR	miR-182	Invasion and migration	PCa	[61]
VIRMA	Stablize lncRNA	LncRNA-CCAT1/CCAT2	Proliferation and invasion	PCa	[62]
METTL3	Stablize lncRNA	LncRNA-SNHG7	Proliferation and glycolysis	PCa	[63]
METTL3	Stablize lncRNA	LncRNA-MALAT1	Proliferation and invasion	PCa	[64]
IGF2BP2	Stablize lncRNA	LncRNA-PCAT6	Bone metastasis	PCa	[66]
METTL3	Promote the maturation of pri-miR	miR-221/222	Proliferation	BCa	[69]
METTL3	Promote the maturation of pri-miR	miR-146a-5p	Apoptosis	BCa	[70]
FTO	Inhibit the maturation of pri-miR	miR-576	Proliferation, invasion, and metastasis	BCa	[71]
METTL3	Stablize lncRNA	LncRNA-NEAT1	Proliferation and migration	RCC	[78]
METTL14	Degrade lncRNA	LncRNA-NEAT1_1	Growth and metastasis	RCC	[80]
METTL14	Degrade lncRNA	Lnc-LSG1	Metastasis	RCC	[81]
YTHDF2	Degrade circRNA	circPOLR2A	Proliferation, migration, invasion, and angiogenesis	RCC	[82]
YTHDC1	Promote transport into cytoplasm	circMET	Proliferation	RCC	[83]

**Table 2:** Molecular mechanism and biological functions of ncRNAs in m6A.

NcRNA	Mechanism	Molecule	Biological function	Tumor	References
CircPDE5A	Block the WTAP-dependent methylation by forming a complex	WTAP	Migration and invasion	PCa	[67]
CircPTPRA	Bind to KH domain of IGF2BP1	IGF2BP1	Proliferation, migration, and invasion	BCa	[72]
miR-5581-3p	Act as a sponge to mRNA	FTO	Migration and proliferation	BCa	[73]
Circ0008399	Promote the formation of WTAP/METTL3/METTL14	WTAP/METTL3/METTL14	Apoptosis and drug-resistance	BCa	[74]
LncRNA-DMDRMR	Act as a cofactor for IGF2BP3	IGF2BP3	Proliferation, invasion, and migration	RCC	[79]
miR-155	Inhibit the expression	FTO	Apoptosis	RCC	[84]
miR-501-3p	Act as a sponge to mRNA	WTAP	Proliferation	RCC	[85]
circPLIN2	Act as a cofactor for IGF2BPs	IGF2BPs	Proliferation, invasion, and migration	RCC	[86]

## The role of m6A regulators interacted with mRNAs in urinary tumors

### The interaction between m6A writers and mRNAs in urinary tumors

Abnormal m6A methylation has been identified in multiple malignancies, and m6A writers play a crucial role in urinary cancers by regulating methylation levels [14]. Cai et al. [87] found that, in PCa, METTL3 silencing can regulate the hedgehog pathway by reducing m6A modification on GLI1, promoting apoptosis. METTL3 can also promote WNT pathway activation by binding to the m6A methylation site on LEF1, promoting PCa progression [88]. Yuan et al. [89] reported that METTL3 promotes PCa cell proliferation, migration, and invasion by regulating MYC transcript m6A levels. In addition, METTL3 can mediate the m6A modification at the A2696 site of USP4 mRNA, promoting USP4 degradation, and leading to increased ubiquitination of the mRNA-binding protein ELAVL. Degradation of ELAVL1 promotes the expression of ARHGDIA, a key migration-related factor, leading to increased invasion and migration of PCa cells [90]. Cotter et al. [91] showed that low-level expression of METTL3 independently upregulated NR5A2 in an androgen receptor signaling pathway, leading to the development of castration-resistant PCa (CRPC).

Tumor-associated macrophages play an important role in the development and progression of cancers. Jia et al. [92] reported that PCa cell-derived lipid A4 can promote M2-like macrophage polarization by inhibiting METTL3. In addition, METTL14 also plays a carcinogenic role in PCa. Wang et al. [93] found that METTL14 promotes the progression of PCa by reducing the expression of THBS1.

In BCa, METTL3 promotes MYC expression by directly targeting AF4/FMR2 family member 4, a key regulatory factor for IKBKB in NFKB signaling, and targeting m6A modification sites in RELA, causing it to bind to MYC promoter sites [94]. Jin et al. [95] showed that METTL3 promotes the translation of ITGA6 by regulating m6A methylation levels in the 3′UTR of ITGA6 mRNA, thus promoting the adhesion, migration, and invasion of BCa cells. Subsequently, Yang et al. [96] found that the m6A site on the 3′UTR of CDCP1 mRNA was preferentially recognized by METTL3 and YTHDF1 to promote CDCP1 translation, thus promoting BCa development. Xie et al. [97] reported that METTL3/YTHDF2 promoted BCa proliferation and metastasis by degrading SETD7 and KLF4 mRNA. Wang et al. [98] found that METTL3-deficient mice had reduced BCa tumorigenesis and tumor angiogenesis, possibly due to decreased TEK and VEGF-A mRNA and protein expression levels. Liu et al. [99] reported that PM2.5 exposure promoted METTL3 expression by reducing METTL3 promoter methylation and increasing the affinity of the nuclear transcription factor HIF1A. Elevated METTL3 leads to BCa progression and angiogenesis by promoting BIRC5 expression. METTL14 expression is low in BCa tumor-initiating cells (TIC). METTL14 knockout promoted proliferation, metastasis, and occurrence of TICs in BCa [100]. Huang et al. [101] further demonstrated that METTL14 inhibits BCa cell migration, invasion, and EMT by modifying the stability of USP38 mRNA through m6A modification.

In RCC, METTL3 plays a dual role. Li et al. [102] showed that patients with high METTL3 expression had significantly better survival than did those with low expression in Caki-1 and Caki-2 cell lines. METTL3 knockdown promoted cell proliferation, and migration, and decreased G0/G1 cell cycle arrest. Shi et al. [103] reported that METTL3 promoted the progression of clear cell RCC (ccRCC) by targeting the translation of ABCD1 through its ATP-binding box. ABCD1 inhibits fatty acids in an m6A-dependent manner, revealing a relationship between m6A modification and tumor metabolic reprogramming. Zhu et al. [104] demonstrated that METTL3 can promote tumor formation in ccRCC by promoting HHLA2 mRNA expression. Wang et al. [105] reported that METTL14 expression in tumor tissue was significantly lower than that of normal tissues, and patients with low METTL14 expression had a worse prognosis. METTL14 deletion led to the accumulation of BPTF, and the BPTF super-enhancer promoted pulmonary metastasis of RCC by increasing the expression of the glycolytic reprogramming proteins ENO2 and SRC [106]. Tang et al. [107] confirmed that WTAP promotes RCC cell proliferation by directly binding CDK2 transcripts, enhancing their stability and increasing expression.

In summary, m6A writers can affect the proliferation, invasion, migration, and drug resistance of urinary tumors by binding to m6A sites on RNAs, impacting downstream signaling pathways, including WNT, c-Myc, NFKB, VEGFA, and others.

### The interaction between m6A erasers and mRNAs in urinary tumors

FTO, the first m6A demethylation enzyme identified, showed that m6A methylation is a dynamic and reversible regulatory process [30]. Subsequently, the ALKBH family, as a group of m6A demethylation enzymes, was found to play an important role in regulating RNA and DNA methylation levels. However, m6A demethylase dysregulation also appears in urinary tumors, indicating that demethylase may regulate the occurrence and development of cancer by affecting downstream target gene expression.

Zhu et al. [108] reported that FTO inhibits PCa cell invasion and migration by reducing overall m6A methylation levels. Li et al. [109] further explored the mechanism, finding that FTO regulates the proliferation, invasion, and migration of PCa by altering the melanocortin 4 receptor expression level. Recently, Zou et al. [110] found that the chloride intracellular channel 4 methylation level increased after FTO knockdown, promoting PCa invasion and metastasis.

In BCa, the deubiquitination enzyme USP18 up-regulated FTO expression at the protein level and facilitated the stability of PYCR1 mRNA in an m6A-dependent manner, thus promoting BCa progression [111]. Tao et al. [112] found that FTO-mediated demethylation of MALAT1 enhanced mRNA stability and thus interfered with miR-384 interaction. This stability increased the expression of AL2, promoting BCa progression. In addition, Yu et al. [113] reported that ALKBH5 inhibits the expression of CK2α mRNA by recognizing the m6A site in the 3′UTR, thus affecting the glycolysis of BCa cells and increasing their sensitivity to cisplatin.

Zhuang et al. [114] found that FTO inhibited the progression of ccRCC by reducing m6A levels in PGC-1α mRNA and promoting its expression, thereby inducing oxidative stress and ROS production. However, Xiao et al. [115] proposed a different view, reporting that FTO was upregulated in VHL-deficient ccRCC and promoted tumor metabolic reprogramming by targeting the glutamine transporter SLC1A5, contributing to the progression and lethality of ccRCC. In addition, high expression of FTO in ccRCC cells with a low expression of HIF2α contributes to the progression of ccRCC through stabilizing bromodomain-containing protein 9 mRNA by acting as m6A demethylase [116]. Shen et al. [117] found that FTO regulates ccRCC growth and migration through the m6A/YTHDF2/PDK1 axis. In addition, FTO promotes the growth and metastasis of ccRCC by affecting autophagy. Xu et al. [118] showed that after silencing FTO, autophagy flux was downregulated through inhibition of ATG5 and ATG7, enhancing the stability of salt-inducible kinase 2 mRNA to promote ccRCC progression. Zhang et al. [119] reported that ALKBH5 promotes the expression of AURKB in an m6A-dependent manner, thus promoting the proliferation, migration, and invasion of ccRCC.

In summary, m6A erasers can affect RNA expression by demethylating RNAs. In urinary system tumors, the main m6A erasers are FTO and ALKBH5, which play a role in the initiation and progression of tumors, affecting tumor proliferation, invasion, migration, drug resistance, glycolytic reprogramming, autophagy, and other malignant behaviors.

### The interaction between m6A readers and mRNAs in urinary tumors

M6A readers determine the functions of downstream signaling pathways regulated by m6A modification. They play crucial roles in regulating tumor biological processes by impacting growth, proliferation, invasion, and metastasis [120–122].

YTHDF2 mediated the degradation of tumor suppressor LHHP and NKX3-1 mRNA in an m6A-dependent manner, and accelerated AKT phosphorylation, leading to the occurrence of PCa [123]. Li et al. [124] found that YTHDF2 increased the proliferation, migration, and invasion of PCa by enhancing TRIM44 expression. The same group [125] subsequently demonstrated that PLK1 can be regulated by YTHDF1 in an m6A-dependent manner, regulating PCa progression by influencing the PI3K/AKT pathway. Yuan et al. [126] found that SLC12A5 complexes with YTHDC1 regulate the expression of HOXB13 through m6A modification, thus promoting PCa progression and drug resistance. In RCC, EGR2 promotes the expression of IGF2BPs, enhancing the stability of S1PR3 mRNA, ultimately affecting the PI3K/AKT pathway, and promoting RCC tumorigenesis [127]. Li et al. [128] reported that the YY1/HDAC2 complex in ccRCC inhibited the expression of YTHDC1, thereby affecting the expression of its downstream target gene ANXA1 and inhibiting the sensitivity of ccRCC to tyrosinase inhibitors.

In summary, the m6A readers YTHDF2, YTHDF1, YTHDC1, and IGF2BPs in urinary tumors affect RNA stability by recognizing m6A methylation sites on RNA, and regulate tumor proliferation, invasion, migration, and drug resistance through PI3K/AKT pathways.

## Conclusions and further considerations

M6A methylation is one of the most common post-transcriptional modifications, and m6A methylation dysregulation is often controlled by its three regulators: writers, readers and erasers [129]. Among them, writers modify m6A methylation by binding to the methylation sites on RNA. Readers bind to the modification sites and determine their downstream functions, including stabilizing or degrading the RNA. Erasers function by dynamically regulating m6A methylation levels and can remove the methylation of RNA [12]. Therefore, the imbalance of these three m6A regulators plays an important role in the occurrence and development of tumors.

M6A regulators not only regulate the methylation level of mRNA and affect downstream protein synthesis but also help regulate ncRNAs [130]. For example, methylation regulators stabilize or degrade the expression of ncRNAs, participate in the splicing and maturation of pri-miRNAs, and impact the transport of ncRNAs from the nucleus to the cytoplasm [3]. Simultaneously, ncRNAs can also influence the methylation level of cells by affecting m6A regulators. For example, miRNAs can act as a sponge for m6A regulators to inhibit the expression of target genes [131].

As a new therapeutic method, tumor immunotherapy has had a profound impact on the outcomes of a variety of cancers. The main function of T cell immune checkpoints is to prevent pathologic T cell overactivation [132]. Programmed cell death 1 (PD-1) is clinically one of the most important immune checkpoints, and the overexpression of PD-1 may be caused by long-term tumor infiltrating. PD-1 binds to programmed cell death ligand 1 (PD-L1) on the surface of tumor cells, leading to the exhaustion of T cells and tumor immune escape [133]. Therefore, it will be of great significance to explore the relationship between m6A modification and tumor immunity. Ni et al. [134] found that METTL3 increases m6A methylation by recognizing the methylation site at the 3′UTR of PD-L1 mRNA, thus promoting PD-L1expression, ultimately leading to the cytotoxicity of BCa cells against CD8+ T cells. Wang et al. [135] showed that removal of the m6A methyltransferases METTL3 and METTL14 enhanced the response to anti-PD-1 therapy in colorectal cancer and melanoma with microsatellite instability. Wan et al. [136] reported that the METTL3/IGF2BP3 pathway enhanced PD-L1 mRNA expression, leading to breast cancer cell immune escape. Liu et al. [137] found that the small-molecule cucurbitacin B could inhibit the recognition of m6A methylation sites by IGF2BP1, thereby blocking the expression of PD-L1 and activating tumor immune regulation. Moreover, METTL3 increased the expression of circIGF2BP3 through m6A modification, and further enhanced the expression of PD-L1 through the miR-328-3p/miR-3173-5p/PKP3/OTUB1 pathway, ultimately leading to immune escape in non-small cell lung cancer [138].

Abnormal m6A methylation levels and expression of ncRNAs are often detected in urinary tumors, which are involved in the occurrence, development, migration, immune escape, drug resistance, and other processes of urinary tumors. Therefore, further study of its pathogenesis is of great significance for the development of specific targeted drugs.
